# Supplementary material for: Single-nucleus transcriptomics reveal the differentiation trajectories of periosteal skeletal/stem progenitor cells in bone regeneration
Source: eLife. 2024 Dec 6;13:RP92519. doi: 10.7554/eLife.92519 (PMC11623931; doi:10.7554/eLife.92519)
Supplement: Supplementary file 1. [file elife-92519-supp1.docx]

| **Score** | **Genes** |
| --- | --- |
| **Stem/progenitor score** | *Ly6a, Cd34, Dpp4, Pi16* |
| **Fibrogenic score** | *Postn, Aspn, Col3a1, Col5a1, Col8a1* |
| **Chondrogenic score** | *Acan, Col2a1, Sox9, Fgfr3* |
| **Osteogenic score** | *Sp7, Alpl, Ibsp, Ifitm5, Bglap* |
| **Notch score** | *Notch1, Notch3, Notch2, Notch4, Jag1, Dtx3, Fbxw7, Psen1, Epn2, Sel1l, Maml1, Spen, Anxa4* |
| **ECM score** | *Col5a1, Col5a2, Col5a3, Col3a1, Col8a1, Col8a2, Col12a1, Eln, Postn, Aspn, Lox, Tll1, Smoc1, Pxdn, Smoc2* |
| **BMP score** | *Bmp5, Bmpr1a, Acvr1, Bmpr2* |
| **TGFb score** | *Tgfb2, Tgfb3, Tgfbr1, Tgfbr2, Acvr1* |
| **PDGF score** | *Pdgfc, Pdgfd, Pdgfra, Pdgfrb* |
| **POSTN score** | *Postn, Itgav, Itgb5* |
| **PTN score** | *Ptn, Sdc2* |
| **ANGPLT score** | *Angptl2, Angptl4, Cdh11, Itga1, Itga5* |
| **Total ligand score** | *Bmp5, Tgfb2, Tgfb3, Postn, Ptn, Angptl4, Angptl2, Pdgfc, Pdgfd* |
| **Total receptor score** | *Bmpr1a, Acvr1, Bmpr2, Tgfbr1, Tgfbr2, Itgav, Itgb5, Sdc2, Ncl, Pdgfra, Pdgfrb* |
