# Supplementary material for: Single-nucleus transcriptomics reveal the differentiation trajectories of periosteal skeletal/stem progenitor cells in bone regeneration
Source: eLife. 2024 Dec 6;13:RP92519. doi: 10.7554/eLife.92519 (PMC11623931; doi:10.7554/eLife.92519)
Supplement: Supplementary file 2. [file elife-92519-supp2.docx]

| **Group of regulons** | **Regulons** |
| --- | --- |
| **Fibro-core regulons** | Arnt, Bach1, Bcl3, Cux1, E2f3, Elk4, Etv6, Fosl2, Foxn3, Foxp1, Jun, Junb, Mdb4, Meis1, Myc, Nfib, Pbx1, Pbx3, Rocor1, Runx1, Six1 |
| **Chondro-core 1 regulons** | Npas2, Hif1a, Sp1, Arntl, Bach2, Nfkb1, Maf, Tead1, Nfatc2 |
| **Chondro-core 2 regulons** | Sox6, Sox5, Sox9, Mef2c, Nfat5, Mef2d, Ccnt2, Clock, Ppard, Irf2, Trp53, Peg3, Creb3l2, Ets1 |
| **Osteo-core regulons** | Nr2f6, Nfic, Shox2, Tbx2, Sp7, Bcl11b, Tcf7, Runx2 |
