# Supplementary material for: Single-nucleus transcriptomics reveal the differentiation trajectories of periosteal skeletal/stem progenitor cells in bone regeneration
Source: eLife. 2024 Dec 6;13:RP92519. doi: 10.7554/eLife.92519 (PMC11623931; doi:10.7554/eLife.92519)
Supplement: Supplementary file 3. [file elife-92519-supp3.docx]

| **Reactome number** | **Term** | **Adjusted p-value** |
| --- | --- | --- |
| R-HSA-157118 | Signaling By NOTCH | 2,52E-04 |
| R-HSA-1912408 | Pre-NOTCH Transcription And Translation | 0,00250164 |
| R-HSA-1912422 | Pre-NOTCH Expression And Processing | 0,00331873 |
| R-HSA-5619507 | Activation Of HOX Genes During Differentiation | 0,00394002 |
| R-HSA-9018519 | Estrogen-dependent Gene Expression | 0,00697148 |
